# Role of Gut Microbiota in Bridging Vitamin D Deficiency and Type 2 Diabetes Mellitus Pathogenesis

**DOI:** 10.3390/microorganisms14030628

**Published:** 2026-03-11

**Authors:** Yinghua Zhan, Jing Liu, Qiannan Di, Lixin Na

**Affiliations:** 1 School of Public Health, Shanghai Jiao Tong University School of Medicine, Shanghai 201318, China; yinghua.zhan@sjtu.edu.cn; 2 The College of Medical Technology, Shanghai University of Medicine and Health Sciences, Shanghai 201318, China; liuj_18@sumhs.edu.cn; 3 College of Public Health, Shanghai University of Medicine and Health Sciences, Shanghai 201318, China; diqiannan@yeah.net

**Keywords:** 25-hydroxyvitamin D, vitamin D, type 2 diabetes mellitus, gut microbiota, microbial metabolites

## Abstract

Type 2 diabetes mellitus (T2DM) is a complex metabolic disorder. The nutritional status of vitamin D, an essential micronutrient, is closely linked to the onset and progression of T2DM. A growing body of research has shown that gut microbiota and its metabolites are emerging as a biological link connecting vitamin D and systemic glucose metabolism. Gut dysbiosis is prevalent in T2DM patients, which is characterized by reduced gut microbial diversity, increased abundance of pathogenic bacteria, and abnormal production of key metabolites such as short-chain fatty acids, bile acids and tryptophan derivatives. These abnormal changes in gut microecology and metabolites can impair intestinal barrier integrity and induce chronic low-grade inflammation in the body, and vitamin D deficiency may further exacerbate these abnormal processes. The evidence suggests that the regulatory effect of vitamin D on systemic glucose metabolism may be partially achieved through gut microbiota-related pathways. This review aims to explore whether, and by what mechanisms, the gut microbiota mediates the regulatory effect of vitamin D on T2DM. It also intends to conduct an analysis of the potential molecular mechanisms underlying the interactions between vitamin D, gut microbiota and T2DM, so as to provide a new theoretical basis and research ideas for the prevention and intervention of T2DM.

## 1. Introduction

Diabetes is a chronic disease marked by hyperglycemia. It has become one of the most important public health challenges worldwide [1]. As of 2024, the global number of adults with diabetes aged 20–79 years reached 589 million. It is projected to rise to 853 million by 2050. Type 2 diabetes mellitus (T2DM) accounts for more than 85% of this number [1,2]. T2DM is driven by insulin resistance (IR), impaired β-cell function and persistent hyperglycemia. Current studies show that the development of T2DM results from long-term interactions between genetic susceptibility and environmental factors [3]. Unhealthy lifestyles, excessive energy intake and micronutrient deficiencies are all regarded as important environmental risk factors [4]. Among the many nutrients, vitamin D is important. A growing body of evidence suggests that vitamin D status is closely linked to insulin resistance and T2DM risk [5,6,7]. The association between vitamin D and T2DM cannot be explained by a single pathway alone. Gut microbiota may play an important role in this association [8]; however, how gut microbiota mediate the effect of vitamin D on T2DM is not yet fully clear.

In T2DM patients, the composition and diversity of gut microbiota change significantly. This suggests that gut microbial dysbiosis may be involved in the development and progression of IR and T2DM [9,10,11]. T2DM patients generally have reduced microbial diversity, an increased proportion of pathogenic bacteria and disrupted microbial metabolic functions. They also have an impaired intestinal barrier and chronic low-grade inflammation [12,13]. Therefore, gut microecology is regarded as an important pathological link in T2DM. Changes in the structure and diversity of gut microbiota can directly or indirectly affect the levels of its functional molecules and metabolites. These include short-chain fatty acids (SCFAs), tryptophan (Trp) derivatives, bile acids (BAs) and branched-chain amino acids (BCAAs) [14]. These gut metabolites can act as key signaling molecules in the host’s metabolism. They regulate insulin sensitivity, glucose homeostasis and immune responses [14,15,16,17]. Hence, regulating gut microbiota composition and restoring the balance of its metabolites to maintain intestinal homeostasis is of great significance for improving T2DM-related metabolic disorders.

Individuals with vitamin D deficiency also exhibit analogous patterns of gut dysbiosis to those seen in patients with T2DM [18,19]. The vitamin D/vitamin D receptor (VD/VDR) signaling pathway not only modulates the expression of antimicrobial peptides, preserves intestinal barrier integrity and impacts mucosal immunity, but also changes the abundance and composition of gut microbiota [8]. This further exerts an indirect effect on the production of microbial metabolites. There is a correlation between vitamin D’s role in ameliorating T2DM and its regulatory effect on gut microbiota. The potential protective effect of vitamin D against T2DM is most likely achieved through its modulation of gut microbiota and their metabolites [7,8,15,20,21,22]. Although numerous studies have independently explored the associations among vitamin D, gut microbiota, and T2DM, comprehensive summaries that conceptualize these three components as an integrated regulatory axis remain scarce, and findings from human, animal, and in vitro studies have yet to be integrated into a unified mechanistic framework [8,19]. Therefore, by summarizing the existing evidence, we found that the gut microbiome can serve as an important mediating factor in the association between vitamin D and T2DM. between vitamin D and T2DM association.

There may be a close connection between vitamin D, gut microbiota, and T2DM, but the mechanism of how vitamin D influences T2DM through the gut microbiota is still not clear. In this review article, we discuss in detail how vitamin D and T2DM interact through intestinal barrier function, inflammation–immune modulation, and microbial metabolite-mediated pathways, thereby affecting the regulation of glucose metabolism.

## 2. Literature Search and Study Selection

This review was conducted as a structured narrative review. PubMed, Web of Science, and Google Scholar databases were searched for reports until 15 December 2025 using the following terms: “vitamin D” OR “25-hydroxyvitamin D” AND “type 2 diabetes mellitus” OR “T2DM”, and “microbiota*” AND “type 2 diabetes mellitus” OR “T2DM”, and “vitamin D” or “25-hydroxyvitamin D” AND “microbiota*”. Studies were included if they: (1) Investigated the relationship between vitamin D and gut microbiota; (2) examined T2DM or metabolic-related outcomes; and (3) were original research articles involving human subjects, animal models, or in vitro experiments. Reviews, conference abstracts, non-English publications, and duplicate records were excluded. Titles and abstracts were screened for relevance, followed by full-text evaluation when necessary. A total of 38 human studies, 22 animal studies, and 14 in vitro studies were included in the final synthesis. Serum 25(OH)D concentrations are uniformly expressed in ng/mL throughout this review (1 ng/mL = 2.5 nmol/L). Study-specific cutoff definitions for vitamin D deficiency or insufficiency were retained as originally reported, acknowledging heterogeneity in threshold criteria across studies.

## 3. Vitamin D Deficiency and Type 2 Diabetes Mellitus Risk

Substantial observational evidence has demonstrated that vitamin D deficiency, defined by reduced serum 25(OH)D levels, is associated with a higher risk of T2DM [23]. A Danish population study involving 222,311 participants reported a significant inverse association between 25(OH)D levels and type 2 diabetes risk, with each 10 nmol/L reduction in 25(OH)D corresponding to a 15% increase in diabetes risk [24]. Consistent findings were reported in the Spanish SUN project, where predicted serum vitamin D levels were negatively correlated with the risk of developing T2DM [25]. Data from NHANES also indicate that optimal 25(OH)D concentrations (~30 ng/mL; originally reported as ~75 nmol/L; 1 ng/mL = 2.5 nmol/L) are associated with the lowest T2DM risk [26].

Observational evidence suggests that vitamin D deficiency is associated with a high risk of T2DM, indicating that vitamin D deficiency is one of the many factors contributing to an increased risk of T2DM. However, evidence from randomized controlled trials (RCTs) remains heterogeneous. Some trials have reported improvements in glycemic parameters. For example, six months of high-dose vitamin D supplementation has been shown to significantly improve peripheral insulin sensitivity and β-cell function [27]. Another RCT with a 30-month intervention period also found that vitamin D supplementation significantly reduced levels of fasting insulin and homeostasis model assessment of insulin resistance (HOMA-IR) in middle-aged and elderly patients with T2DM [28]. Nevertheless, a recent large-scale trial in older adults found no significant reduction in incidences of T2DM with vitamin D supplementation, although pooled analyses across multiple RCTs suggested a modest protective effect [29]. Overall, findings from RCTs are inconsistent. This is probably because of differences in supplementation dose, baseline vitamin D status, ethnicity and participants’ metabolic characteristics. People with near-normal baseline 25(OH)D usually get little benefit from supplementation. In contrast, those with severe vitamin D deficiency are most likely to show a response [21,29,30,31].

Despite consistent evidence from various study designs indicating a strong association between vitamin D status and the risk of T2DM, classical endocrine pathways alone are insufficient to fully explain its metabolic effects. Increasing research suggests that the regulatory role of vitamin D on insulin sensitivity and glucose homeostasis may be partly mediated through its influence on the gut microbiota and their derived metabolites [8], providing new theoretical support for a potential vitamin D–gut microbiota–T2DM regulatory axis.

## 4. Vitamin D and Gut Microbiota in Type 2 Diabetes Mellitus

### 4.1. Effects of Vitamin D on Gut Microbiota Composition and Diversity

An increasing body of evidence suggests that vitamin D status is closely associated with gut microbiota diversity and composition (Table 1). Observational studies have shown that in infants, those with vitamin D deficiency exhibited significantly lower Pielou’s evenness and Shannon diversity at six months compared with vitamin D-sufficient infants, indicating that vitamin D deficiency may restrict the establishment and development of early intestinal microbiota diversity [32]. In postmenopausal women, the Observed, Chao1, and ACE indices in a low vitamin D level group were all lower than those in a high vitamin D level group, suggesting that low vitamin D status is associated with decreased intestinal microbiota richness. At the level of microbiota composition, the high vitamin D (HVD) group had enriched abundances of *Christensenellaceae*, *Eggerthellaceae*, and *Cloacibacillus* in the intestine, while the low vitamin D (LVD) group was dominated by *Bifidobacterium*, *Gemella*, and *Lachnoclostridium* [33]. Studies on obese populations have found that individuals with optimal serum 25(OH)D levels had significantly higher intestinal microbiota diversity than those with low levels, and there were intergroup differences in the relative abundances of *Bacteroides*, *Prevotella*, and some *Clostridium* species [34]. Therefore, the aforementioned observational studies across different populations indicate that low vitamin D status is closely associated with reduced gut microbiota diversity and dysbiosis.

Interventional studies further support the association between vitamin D and gut microbiota. In healthy individuals, moderate-dose vitamin D supplementation significantly increased the microbial abundance of *Gemella*, *Fournierella*, and *Ruminococcaceae UCG-010*, while the percentage change in 25(OH)D was negatively correlated with gut microbial stability [35]. In healthy women with vitamin D deficiency, vitamin D supplementation not only significantly enhanced gut microbial diversity but also increased the ratio of *Bacteroidetes* to *Firmicutes* and promoted the enrichment of beneficial probiotics such as *Akkermansia* and *Bifidobacterium* [36]. In specific populations, in a trial involving vitamin D-deficient overweight or obese adults, vitamin D supplementation increased the abundance of *Granulicatella* in the gut of the participant, accompanied by a decrease in the abundance of *Ruminococcus* [37]. Yet, not all results were positive [38,39].

Animal studies further indicate that vitamin D supplementation increases *unclassified Muribaculaceae* and *Lachnospiraceae_NK4A136_group*, while reducing the relative abundance of *Lactobacillus* and *Odoribacter* in KKay mice [40]. Particularly in a high-fat diet-induced dysbiosis mouse model, vitamin D supplementation increased the relative abundance of *Prevotella* and *Porphyromonadaceae*, while reducing *Mucispirillum*, *Acetatifactor*, *Desulfovibrio*, and *Oscillospira*, thereby alleviating dysbiosis. This was accompanied by a reduced *Firmicutes*/*Bacteroidetes* (F/B) ratio and enhanced α-diversity, thereby improving the dysbiotic state induced by a high-fat diet [41,42].

Combining the results of diverse studies, vitamin D supplementation is associated with the growth of SCFA-producing bacteria (e.g., *Anaerostipes*, *Akkermansia*) in the intestine and reduces the abundance of lipopolysaccharide (LPS)-positive bacteria (e.g., *Escherichia*–*Shigella*, *Desulfovibrio*). Other microbial taxa may exhibit heterogeneous responses, potentially influenced by baseline vitamin D status, metabolic condition, supplementation dose and duration, as well as differences in sequencing and analytical methodologies. Since SCFA-producing bacteria can maintain intestinal barrier integrity by producing propionate and butyrate, while LPS-positive bacteria may induce intestinal inflammation, vitamin D may thus improve the host’s intestinal microecological environment and metabolic status by regulating the balance of intestinal microbiota. These may also provide a potential mechanistic explanation for the protective effect of vitamin D in metabolic-related diseases; however, further well-designed studies are required to clarify the context-dependent nature and causality of these associations.

**Table 1:** Gut microbiota changes in relation to vitamin D status or supplementation.

Study	Study Design	Subjects/Model	Vitamin D Exposure	Microbial Taxa (↑/↓)
Song et al. [32]	Cohort study	87 mother–infant pairs VD deficient (*n* = 59) and VD sufficient (*n* = 28). Infant stool samples at 1 month (M1) and 6 months (M6)	Maternal VD insufficiency vs. sufficiency	M1: *Campylobacter* ↓; *Bacteroides* ↑. M6: *Clostridium* and *Morganella* ↑; *Epulopiscium* and *Proteus* ↓.
Gong et al. [33]	Cross-sectional study	88 postmenopausal women HVD (*n* = 44) and LVD group (*n* = 44)	HVD group with 25(OH)D levels ≥ 20 ng/mL and a LVD group with 25(OH)D levels < 20 ng/mL	In the HVD group: *Christensenellaceae*, *Eggerthellaceae*, *Cloacibacillus* ↑ In the LVD group: *Bifidobacterium*, *Bacillus*, *F0332*, *Jeotgalibaca*, *Lachnospiraceae (unclassified)*, *Lachnospira (UC5_1_2E3 group)*, *Ruminococcus gnavus group* ↑.
Boughanem et al. [34]	A nested cross-sectional and prospective study	91 adults with obesity and metabolic syndrome Optimal 25(OH)D (*n* = 45) and Low 25(OH)D (*n* = 46)	Optimal 25(OH)D > 24.05 ng/mL Low 25(OH)D ≤ 24.05 ng/mL	Baseline differences in Low 25(OH)D group: *Bacteroides*, *Prevotella*, *Clostridiales feature 1*, *Clostridiales feature 2* ↑.
Wyat et al. [35]	RCT	Vitamin D_3_ group (*n* = 20) vs. Placebo group (*n* = 21)	Vitamin D_3_ group: 4000 IU/day for 12 weeks Placebo group: Matching placebo for 16 weeks.	In Vitamin D_3_ group: *Bifidobacterium*, *Firmicutes*, *Anaerostipes*, *Erysipelotrichaceae UCG-003* ↑; *Bacteroides*, *Faecalibacterium*, *Prevotella*, *Eubacterium coprostanoligenic* ↓.
Naderpoor et al. [37]	RCT	Overweight/obese, vitamin D-deficient adults (25(OH)D ≤ 20 ng/mL) Vitamin D group (*n* = 14) Placebo group (*n* = 12)	Vitamin D group: 100,000 IU cholecalciferol, followed by 4000 IU/day for 16 weeks Placebo group: Matching placebo for 16 weeks	After supplementation: *Lachnospira* ↑ and *Blautia* ↓. In participants achieving higher vitamin D status (>75 nmol/L): *Coprococcus* ↑ and *Ruminococcus* ↓.
Singh et al. [36]	Single-arm study	Healthy vitamin D-deficient women (*n* = 80)	Received 50,000 IU of oral vitamin D_3_ weekly for 12 weeks.	*Akkermansia*, *Bifidobacterium*, *Bacteroidetes*/*Firmicutes* ratio and gut microbial diversity ↑.
Zhang et al. [40]	Animal study	KKay mice	Weekly intraperitoneal Vitamin D_3_ at different doses vs. PEX168 vs. vehicle	After Vitamin D_3_ supplementation: *Muribaculaceae (unclassified)*, *Lachnospiraceae_NK4A136_group* ↑. *Lactobacillus*, *Odoribacter* ↓.
Zhang et al. [41]	Animal study	Rats with HFD-induced NAFLD	Vitamin D injection, twice weekly, 12 weeks	After Vitamin D supplement: α-diversity, *Prevotella*, *Porphyromonadaceae* ↑. *Firmicutes*/*Bacteroidetes* ratio, *Mucispirillum*, *Acetatifactor*, *Desulfovibrio*, *Oscillospira* ↓.
Xiang et al. [42]	Animal study	Male C57BL/6J mice with HFD-induced obesity	Dietary Vitamin D_3_ supplementation, 5650–11,300 IU/kg, 8 weeks	After Vitamin D_3_ supplementation: *Bacteroidetes*, *Proteobacteria*, *Desulfobacterota*, *Dehalobacterota*, *Odoribacterota*, *Parabacteroides* and α-diversity ↑ *Firmicutes*, *Ruminococcus* and *Firmicutes*/*Bacteroidetes* ratio ↓.
Liu et al. [43]	Animal study	Rats with early-life (0–8 weeks) VD deficiency	Vitamin D deficiency from 0 to 8 weeks (F1), normal diet afterwards; F2 fed normally	In Vitamin D deficiency group: *Desulfovibrio*, *Roseburia*, *Ruminiclostridium*, *Lachnoclostridium*, *A2*, *GCA-900066575*, *Peptococcus*, *Lachnospiraceae_FCS020_group*, *Bilophila* ↑ *Blautia* ↓.

Microbial taxa show genera, species, or families with significant changes. “↑” indicates increased abundance, “↓” indicates decreased abundance.

### 4.2. Gut Microbiota Alterations in Type 2 Diabetes Mellitus

A growing body of evidence indicates that patients with T2DM exhibit significant alterations in gut microbiota composition compared with healthy individuals (Table 2). Overall, it has been frequently reported that the relative abundance of *Firmicutes* increases and *Bacteroidetes* decreases in individuals with prediabetes and T2DM, leading to an elevated F/B ratio, a feature linked to insulin resistance [44,45,46]. At the genus and species levels, gut microbial biomarkers are often altered in T2DM patients. For instance, the abundances of *Bacteroides*, *Methanobrevibacter*, *Paraprevotella* and *Halomonadaceae* are reduced, while *Prevotella*, *Megasphaera*, *Ligilactobacillus* and *Lachnoclostridium* are increased [47]. Additional metagenomic studies across multiple countries have shown notable enrichment of *Clostridium citroniae*, *Enterocloster bolteae* and *Escherichia coli* in T2DM patients. By contrast, *Coprococcus eutactus* and *Ruminococcus sanguinis* are found in significantly lower levels in these patients [48]. Such microbial changes appear to shift progressively from the normoglycemic state to prediabetes and overt T2DM. This suggests these taxa may act as potential early indicators for T2DM onset. In addition to compositional alterations, gut dysbiosis in patients with T2DM may influence metabolic regulation through multiple interconnected pathways. Changes in microbial communities have been linked to impaired intestinal barrier integrity, altered immune–inflammatory responses, and dysregulated production of key microbial metabolites. These alterations are thought to influence insulin signaling and glucose metabolism, potentially contributing to the onset and progression of T2DM [48,49,50,51].

Ethnicity, geographic region, and dietary differences are important factors contributing to the heterogeneity of gut microbiota research findings in T2DM. Microbiome profiles exhibit significant regional variation. Asian T2DM patients exhibit increased abundances of *Bifidobacterium*, *Streptococcus*, and *Prevotella*, while *Bacteroides*, *Faecalibacterium*, and *Blautia* abundance is reduced [52]. In contrast, American T2DM patients exhibit relatively higher abundances of *Bacteroides*, *Faecalibacterium*, and *Blautia*, alongside lower *Alistipes* abundance [53]. In Finnish populations, the enrichment of *Clostridium citroniae*, *Enterocloster bolteae*, *Tyzzerella*, and *Ruminococcus gnavus* were consistently associated with T2DM [48,49]. Additionally, several population-specific studies have reported a decreased *Firmicutes*/*Bacteroidetes* (F/B) ratio, which further confirms the regulatory effects of dietary patterns and population backgrounds on gut microbiota characteristics [54,55]. Moreover, obesity, as the most common comorbidity of T2DM, can independently modulate the patterns of gut microbiota dysbiosis. A German study demonstrated that T2DM patients with comorbid obesity only exhibited mild enrichment of *Escherichia*–*Shigella*. On the contrary, in non-diabetic obese individuals, the abundances of gut bacterial genera such as *Akkermansia*, *Faecalibacterium*, *Oscillibacter*, and *Alistipes* were significantly reduced [56]. These findings suggest distinct dysbiosis patterns between obesity alone and obesity-related T2DM.

Collectively, despite some heterogeneity among studies, converging evidence supports the existence of pronounced alterations in the gut microbiota of T2DM patients, potentially emerging even before clinical onset. Host dietary habits, genetic background, and environmental exposures collectively shape these disease-specific microbial signatures.

**Table 2:** Summary of gut microbiota alterations reported in type 2 diabetes mellitus.

Study	Study Subjects/Country	Microbial Taxa	Change in T2DM (↑/↓)
Wu et al. [52]	3378 healthy individuals and 551 T2DM patients from six Asian studies	ET-L: *Escherichia fergusonii*, *Collinsella aerofaciens*, *Enterococcus faecalis*, *Bifidobacterium longum*. ET-P: *Escherichia fergusonii*, *Megasphaera elsdenii*, *Oscillibacter valericigenes*.	↑
		ET-L: *Phocaeicola vulgatus*, *Bacteroides uniformis*, *Faecalibacterium prausnitzii* ET-P: *Bacteroides koreensis*, *Faecalibacterium prausnitzii*.	↓
Park et al. [53]	1039 T2DM patients and 872 healthy controls from the United States	*Enterocloster bolteae*, *Faecalicatena fissicatena*, *Clostridium symbiosum*, *Faecalibacterium prausnitzii*.	↑
		*Bacteroides koreensis*, *Oscillibacter ruminantium*, *Bacteroides uniformis*, *Blautia wexlerae*.	↓
Ruuskanen et al. [49]	5572 healthy individuals, including 432 who developed T2DM during follow-up, from Finland.	*Clostridium citroniae*, *Enterocloster bolteae*, *Tyzzerella nexilis*, *Ruminococcus gnavus*.	↑
		*Alistipes* spp.	↓
Larsen et al. [55]	18 T2DM patients and 18 non-diabetic individuals from Denmark	*Bacteroides–Prevotella group*, *Lactobacillus group*, *Escherichia*–*Shigella*.	↑
		*Faecalibacterium prausnitzii*, *Roseburia*, *C. coccoides–E. rectale group*.	↓
Thinghol et al. [56]	633 lean individuals without diabetes, 494 obese individuals without diabetes, and 153 obese individuals with T2DM from Germany	*Escherichia*–*Shigella*.	↑
Mei et al. [48]	8117 individuals from 10 cohorts in the USA, Europe, Israel, and China (T2DM: *n* = 1851; prediabetes: *n* = 2770; normoglycemia: *n* = 2277)	*Enterocloster bolteae*.	↑
		*Coprococcus eutactus*, *Turicibacter sanguinis*, *Ruminococcus lactaris*, *Bacteroides plebeius*, *Butyrivibrio crossotus*.	↓
Doumatey et al. [57]	98 T2DM patients and 193 controls from Africa	*Prevotella*, *Peptostreptococcus*, *Desulfovibrio piger*, *Eubacterium*.	↑
		*Clostridiaceae*, *Peptostreptococcaceae*, *Clostridium butyricum*, *Ruminococcus lactaris*, *Anaerostipes*, *Cellulosilyticum ruminicola*.	↓
Qin et al. [9]	71 T2DM patients and 74 healthy controls from China	*Bacteroides caccae*, *Clostridium hathewayi*, *Clostridium ramosum*, *Clostridium symbiosum*, *Eggerthella lenta* and *Escherichia coli*, *Akkermansia muciniphila*, and *Desulfovibrio*.	↑
		*Clostridiales* sp. *SS3/4*, *Eubacterium rectale*, *Faecalibacterium prausnitzii*, *Roseburia intestinalis*, and *Roseburia inulinivorans*.	↓
Alvarez-Silva et al. [58]	279 Danish individuals (138 normoglycemic, 141 T2DM) and 294 Indian individuals (137 normoglycemic, 157 T2DM)	*Lachnoclostridium*.	↑
		*Subdoligranulum*, *Butyricicoccus*, *Anaerosporobacter*.	↓
Karlsson et al. [59]	European women with T2DM (*n* = 53), IGT (*n* = 49), NGT (*n* = 43)	*Lactobacillus gasseri*, *Streptococcus mutans*.	↑
		*Roseburia*, *Eubacterium eligens*, *Bacteroides intestinalis*, *Coriobacteriaceae*.	↓
Morsy et al. [60]	10 T2DM patients and 10 non-diabetic individuals from Egypt	*Bacteroides*, *Blautia*, *and Lachnospiraceae_FCS020_group*.	↑
		*Faecalibacterium* and *Roseburia*.	↓
Letchumanan et al. [61]	45 T2DM patients and 45 non-T2DM individuals from Malaysia	*Escherichia–Shigella*.	↑
		*Anaerostipes* and *Romboutsia*.	↓

Change in T2DM: Relative abundance of taxa in T2DM patients compared with healthy controls, “↑”: Increased; “↓”: Decreased.

### 4.3. Evidence for the Potential Regulatory Axis of Vitamin D Modulating Type 2 Diabetes Mellitus via Gut Microbiota

Accumulating evidence indicates that the influence of vitamin D on T2DM may extend beyond its classical roles in insulin secretion, insulin sensitivity, and calcium homeostasis, and may partly rely on its regulatory effects on the gut microbiota [8]. Dysbiosis of the gut microbiota is recognized as a typical hallmark of T2DM. It is characterized by a reduction in the abundance of *Bifidobacterium*, *Akkermansia*, *Anaerostipes*, and *Lactobacillus*, alongside elevated levels of *Escherichia*–*Shigella*, *Enterococcus*, *Desulfovibrio*, and *Enterobacter*. These changes may be accompanied by an elevated F/B ratio [9,52,59,61,62,63]. Vitamin D deficiency has been associated in some studies with similar microbial shifts, including decreased abundance of *Bifidobacterium*, *Akkermansia*, and *Anaerostipes*, increased abundance of *Desulfovibrio* and *Escherichia*–*Shigella*, and an increased F/B ratio [8,35,36,41,64,65,66,67]. Therefore, the intestinal barrier injury and microbiota imbalance caused by vitamin D deficiency are highly consistent with the microbiota dysbiosis commonly observed in T2DM patients. This suggests that vitamin D may participate in the pathogenesis and progression of T2DM through the pathway of regulating the gut microbiota.

Direct evidence demonstrating that vitamin D reduces the risk of T2DM through gut microbiota-mediated effects (including human cohort mediation analyses, causal inference studies, mechanistic fecal microbiota transplantation experiments, and VDR-targeted intervention studies) remains limited. To date, insufficient evidence is available to confirm the causal and mediating relationships among these three factors. One animal experiment has provided relatively direct clues. In KKay mice, vitamin D supplementation not only improved glucose metabolism, restored the expression of intestinal tight junction proteins, and reduced metabolic endotoxemia and inflammation, but also produced metabolic benefits that could be transferred via fecal microbiota transplantation [40]. This strongly supports the potential mediating role of gut microbiota in the regulation of T2DM by vitamin D. In terms of supportive evidence, a human study found that individuals with low serum 25(OH)D levels (<32 ng/mL) exhibited a typical T2DM-associated gut microbiota dysbiosis. In contrast, those with higher serum 25(OH)D levels showed a more balanced gut microbiota structure, characterized by increased abundance of butyrate-producing bacteria and decreased abundance of pro-inflammatory bacteria [63]. An animal experiment further confirmed that early-life vitamin D deficiency can induce the remodeling of gut microbiota structure in rats, and the variation in the abundance of *Blautia* is significantly correlated with glucose tolerance indices in adult rats. These findings suggest that the metabolic effects of vitamin D deficiency are microbiota-dependent and may have potential intergenerational effects [43]. Meanwhile, another mechanistic study indicated that vitamin D may alleviate T2DM-related metabolic disorders by regulating the gut microbiota–endocannabinoid system signaling pathway [68]. In summary, these studies reveal that the gut microbiota likely serves as a mediator in the effects of vitamin D on T2DM progression. Understanding this regulatory axis provides a useful perspective on nutrition and opens new doors for treating metabolic diseases. The mechanism through which Vitamin D regulates T2DM via gut microbial modulation attracts increasing attention.

## 5. Potential Mechanisms Linking Vitamin D, Gut Microbiota, and Type 2 Diabetes Mellitus

**Figure 1:**
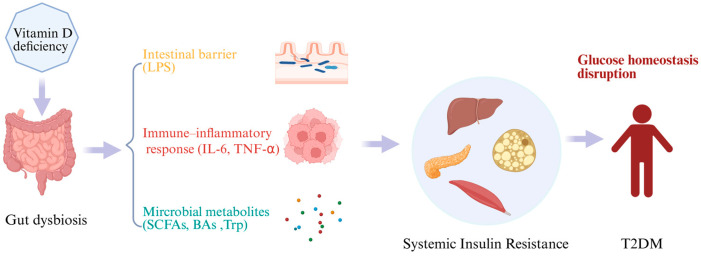
Proposed mechanisms linking vitamin D deficiency, gut microbiota dysbiosis, and type 2 diabetes mellitus. Above is a schematic illustration of the proposed mechanisms by which vitamin D deficiency may contribute to the development of type 2 diabetes mellitus (T2DM) through alterations in the gut microbiota. Vitamin D deficiency is associated with gut dysbiosis, characterized by reduced microbial diversity and compositional imbalance. This dysbiotic state may impair intestinal barrier integrity, leading to increased lipopolysaccharide (LPS) translocation and metabolic endotoxemia. Consequently, activation of immune–inflammatory pathways, including elevated pro-inflammatory cytokines such as interleukin-6 (IL-6) and tumor necrosis factor-α (TNF-α), may promote systemic low-grade inflammation. In parallel, alterations in gut microbiota-derived metabolites, including short-chain fatty acids (SCFAs), bile acids (BAs), and tryptophan (Trp) metabolites may further disturb host metabolic regulation. These combined effects impair insulin signaling in major insulin-sensitive organs, including the liver, skeletal muscle, adipose tissue, and pancreatic β cells, leading to systemic insulin resistance. Persistent insulin resistance ultimately results in glucose homeostasis disruption and facilitates the development of T2DM.

By reviewing current studies, it appears that gut microbiota may act as an intermediary and play an important role in how vitamin D influences the onset and progression of T2DM. In this section, we summarize the potential mechanisms by which vitamin D may regulate T2DM through the gut microbiota from three aspects: the intestinal barrier, immune–inflammatory responses, and microbial metabolites (Figure 1).

### 5.1. Intestinal Barrier Function and Endotoxin Translocation

Vitamin D activates its receptor VDR. It promotes the expression of tight junction proteins, such as occludin, claudin-1 and ZO-1, to strengthen the physical barrier between epithelial cells [69]. It also upregulates the secretion of antimicrobial peptides (e.g., cathelicidin and β-defensin) in intestinal epithelial cells. These peptides kill pathogenic bacteria and inhibit the overproliferation of opportunistic pathogens [70]. In this dual way, vitamin D maintains the integrity of the intestinal epithelial barrier and the homeostasis of intestinal flora. Vitamin D deficiency reduces defensin production, and increases intestinal permeability as a result. This allows intestinal bacteria and their metabolites, especially lipopolysaccharide (LPS), to cross the epithelial barrier and enter the circulatory system more easily [68,71]. Once in the system, LPS binds to lipopolysaccharide-binding protein (LBP). It then activates the Toll-like receptor 4 (TLR4)/myeloid differentiation factor 2 (MD2) complex on the surface of immune cells. This triggers the nuclear translocation of the nuclear factor kappa B (NF-κB) pathway [72]. Consequently, pro-inflammatory cytokines such as tumor necrosis factor-α (TNF-α) and interleukin-6 (IL-6) are released. These cytokines not only directly inhibit the phosphorylation of insulin receptor substrates and block insulin signal transduction. They also damage the function of pancreatic β-cells. Eventually, insulin resistance is induced [22]. LPS translocation caused by barrier damage is recognized as a critical early event linking intestinal microecological imbalance to metabolic disorders [73]. Low vitamin D levels are associated with elevated serum LPS levels. Supplementing vitamin D can reduce serum LPS and pro-inflammatory cytokine levels in T2DM patients. It can also improve their insulin sensitivity [40,42,63,74]. Therefore, LPS translocation is often regarded as a key factor in T2DM-related inflammation and insulin resistance. Its important role in the axis linking vitamin D-modulated intestinal flora to T2DM deserves close attention.

### 5.2. Immune Modulation and Inflammatory Regulation

Vitamin D modulates gut microbiota composition by regulating the body’s immune system. It also regulates immune and inflammatory responses in a reverse manner when gut microbiota status is altered [75]. These regulatory actions go on to affect how type 2 diabetes mellitus develops and progresses. Patients with T2DM usually have chronic low-grade inflammation. This condition is marked by higher levels of pro-inflammatory factors like TNF-α, IL-6 and C-reactive protein [76]. These inflammatory mediators interfere with insulin signaling pathways.

They damage post-receptor signaling and reduce the body’s uptake and use of glucose. Eventually, this leads to insulin resistance [77,78]. An imbalance in immune cell subsets is a major cause of worsening inflammation. This kind of imbalance disrupts the immune homeostasis of the intestinal mucosa. It also directly shows that intestinal homeostasis and systemic immune–inflammatory responses are closely connected [51]. Vitamin D has two key effects on immune and inflammatory responses, and this is mainly achieved by regulating gut microbiota. On the one hand, the VD/VDR signaling pathway can help probiotics like *Bacteroides* and *Lactobacillus* colonize the gut. It also prevents opportunistic pathogens from overgrowing, which in turn maintains the stability of the gut’s microecological balance [35,79]. Restoring gut homeostasis not only eases local and systemic inflammation, but can also adjust glucose metabolism by reducing insulin resistance [51]. On the other hand, this signaling pathway is involved in controlling the differentiation and function of immune cells. When the pathway is activated, it promotes the production of regulatory T cells (Tregs) and prevents pro-inflammatory cell subsets such as Th1 and Th17 from being overactivated [80]. This maintains the Th17/Treg immune balance. This balance is essential for keeping the intestinal mucosal homeostasis intact [51]. When intestinal homeostasis is well maintained, the body produces more SCFAs. SCFAs, especially butyrate, can enhance the function of Tregs and increase the expression of anti-inflammatory genes [81,82]. It does this by maintaining the right ratio of Th17/Treg cells and promoting SCFA production. These actions then have an impact on glucose metabolism. In contrast, low vitamin D levels often lead to gut dysbiosis [18]. This is usually accompanied by endotoxemia and more severe inflammation. It impairs TNF-α-induced TLR4/NF-κB signaling and disrupts the PI3K/AKT insulin signaling pathway [22]. Vitamin D lessens these inflammatory processes by inhibiting NF-κB and the NOD-like receptor family pyrin domain-containing 3 (NLRP3) inflammasome. This reduction then brings down the levels of IL-6, TNF-α and other inflammatory mediators [42]. Therefore, these findings show that vitamin D and gut microbiota work together to regulate immune responses. These joint regulatory effects ultimately affect the health of the body’s glucose metabolism.

### 5.3. The Mediating Role of Microbial Metabolites in the Vitamin D–T2DM Axis

**Figure 2:**
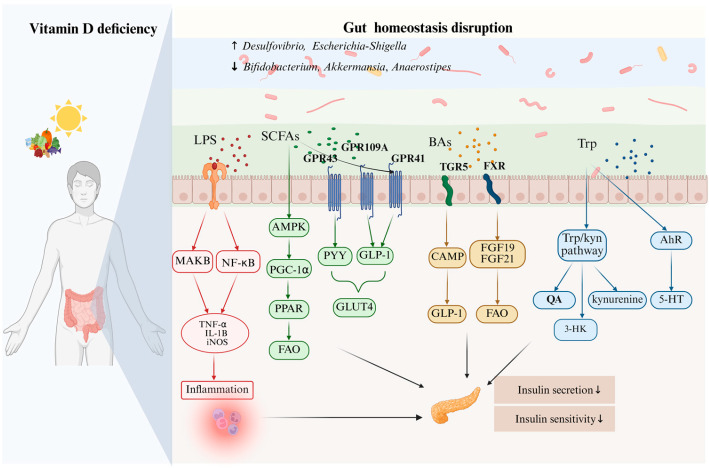
Mechanistic links between vitamin D deficiency, gut microbiota dysregulation, and insulin resistance. Vitamin D deficiency, arising from inadequate sunlight exposure or insufficient dietary intake, has been associated in some studies with shifts in gut microbiota composition, including reduced levels of *Bifidobacterium*, *Akkermansia*, and *Anaerostipes* and increased abundance of *Desulfovibrio* and *Escherichia-Shigella.* Dysbiosis compromises gut barrier integrity and promotes lipopolysaccharide (LPS) translocation, activating MAPK and NF-κB pathways and increasing pro-inflammatory cytokines (TNF-α, IL-1β, iNOS), leading to systemic inflammation. Reduced short-chain fatty acids (SCFAs) weaken GPR43/GPR41/GPR109A signaling, resulting in decreased PYY, GLP-1, and GLUT4 expression, AMPK–PGC-1α–PPAR activation, and fatty acid oxidation (FAO). Altered bile acid (BA) composition impairs TGR5 and FXR signaling, lowering CAMP, FGF19, FGF21, and GLP-1 responses. Disrupted tryptophan (Trp) metabolism shifts toward the kynurenine pathway, increasing kynurenine, QA, and 3-HK while reducing 5-HT and AhR activation. Abbreviations: SCFAs, short-chain fatty acids; BAs, bile acids; LPS, lipopolysaccharide; MAPK, mitogen-activated protein kinase; NF-κB, nuclear factor κB; TNF-α, tumor necrosis factor-α; IL-1β, interleukin-1β; iNOS, inducible nitric oxide synthase; AMPK, AMP-activated protein kinase; PGC-1α, peroxisome proliferator-activated receptor gamma coactivator-1α; PPAR, peroxisome proliferator-activated receptor; FAO, fatty acid oxidation; PYY, peptide YY; GLP-1, glucagon-like peptide-1; BA, bile acid; CAMP, cathelicidin antimicrobial peptide; FGF19/FGF21, fibroblast growth factor 19/21; Trp, tryptophan; QA, quinolinic acid; 3-HK, 3-hydroxykynurenine; 5-HT, serotonin; AhR, aryl hydrocarbon receptor.

Gut microbiota metabolites, such as SCFAs, BAs and Trp, play a key role in regulating systemic glucose metabolism [50,83]. They may also act as mediators in the potential regulatory axis. This axis connects vitamin D, gut microbiota and T2DM regulation. Disturbance to this axis does more than reduce the production of beneficial metabolites like SCFAs. It also promotes the buildup of harmful substances such as LPS. Furthermore, it directly damages the integrity of the intestinal barrier and may cause intestinal permeability [35,68]. This subsequently triggers chronic low-grade inflammation and insulin resistance, ultimately accelerating the pathological progression of T2DM. Accordingly, these metabolites serve as the central hub that links the interplay between vitamin D, gut microbiota and T2DM (Figure 2).

SCFAs include acetate, propionate and butyrate. They are key metabolites produced by intestinal flora through dietary fiber fermentation. Vitamin D binds to its receptor VDR to upregulate the expression of mucins (MUC2, MUC3) and tight junction proteins (ZO-1, occludin) in intestinal epithelial cells. This process enhances the integrity of the intestinal mucosal barrier [69]. It also inhibits the release of intestinal pro-inflammatory factors. In this way, the intestinal immune microenvironment is improved [70]. These changes help maintain intestinal microecological homeostasis. They create a favorable ecological niche for SCFA-producing bacteria such as *Faecalibacterium prausnitzii*, *Roseburia* and *Bifidobacterium* to survive stably and exert their metabolic functions [84,85]. SCFAs can activate multiple G protein-coupled receptors (GPRs), including GPR41, GPR43 and GPR109A. They synergistically regulate energy metabolism and inflammatory responses in the intestine, adipose tissue and immune system [86]. In adipose tissue, GPR41 activation alleviates lipotoxicity. In the immune system, GPR109A activation helps inhibit macrophage-mediated chronic inflammation [87]. Thus, SCFAs participate in regulating the development and progression of T2DM. When propionate activates GPR41/GPR43 on intestinal L cells, they stimulate the secretion of glucagon-like peptide-1 (GLP-1) and peptide YY (PYY). GLP-1 promotes insulin secretion and improves insulin sensitivity. PYY helps suppress appetite [65,88]. Propionate is linked to methylation at the cg26345888 locus, which subsequently suppresses the expression of disabled homolog 1 (DAB1) associated with vitamin D deficiency. This could constitute a propionate-mediated vitamin D epigenetic regulatory pathway, influencing the progression of diabetes [89]. Butyrate stands out for its role in improving immune responses. It can regulate the number and function of regulatory T cells. This reduces inflammation in adipose tissue. It also promotes GLP-1 production, thereby enhancing insulin sensitivity. Meanwhile, butyrate serves as the primary energy source for colonic epithelial cells [86,90]. Furthermore, a synergistic interaction may exist between butyrate and vitamin D signaling. Butyrate inhibits histone deacetylases (HDACs). This enhances histone acetylation at the VDR gene promoter region. It then promotes VDR transcription and protein expression, indirectly boosting VDR activity [91]. On the other hand, vitamin D binds to VDR to promote the differentiation of Treg and inhibit pro-inflammatory factor release [79,91,92]. The two may form a positive feedback loop that jointly maintains immune homeostasis and metabolic balance in the body and plays a regulatory role in the development of T2DM.

Bile acids, including primary and secondary bile acids, are important signaling molecules that link hepatic metabolism, gut microbiota, and host energy homeostasis [93]. Vitamin D exerts a multi-level regulatory effect on bile acid metabolism. When vitamin D binds to VDR, it upregulates the expression of small heterodimer partner (SHP) in the liver. SHP then interacts with hepatocyte nuclear factor 4α, a key protein that controls how bile acids are made. This interaction slows down the activity of cholesterol 7α-hydroxylase (CYP7A1). CYP7A1 is the main enzyme that limits how much bile acid the body produces [94,95]. Activation of the VD/VDR axis also elevates the expression of bile acid efflux transporters, including the bile salt export pump (BSEP) and multidrug resistance-associated protein 2 (MRP2). Meanwhile, it modulates the expression of intestinal bile acid transporters such as the apical sodium-dependent bile acid transporter (ASBT) and organic solute transporter α/β (OSTα/β). These coordinated regulatory changes contribute to the maintenance of bile acid enterohepatic circulation [93,95]. Beyond its direct modulation of host bile acid synthesis and transport, vitamin D indirectly influences bile acid transformation by enhancing intestinal barrier integrity and sustaining gut microbial homeostasis [96]. Adequate vitamin D levels in the body promote the enrichment of commensal bacteria, including *Ruminococcus* and *Eubacterium* species [33]. This bacterial enrichment is accompanied by increased production of bile salt hydrolase (BSH) and enhanced activity of 7α-dehydroxylase. Such alterations accelerate the deconjugation and dehydroxylation of bile acids, thereby facilitating the generation of secondary bile acids like lithocholic acid (LCA) [97]. Altered bile acid composition not only modulates enterohepatic metabolic homeostasis but also regulates metabolic signaling via specific bile acid receptors [93]. Secondary bile acids, particularly LCA, can activate the Takeda G protein-coupled receptor 5 (TGR5) receptor on intestinal L cells, which further triggers the secretion of GLP-1. TGR5 activation in pancreatic β cells also enhances glucose-dependent insulin secretion [98]. Moreover, TGR5 signaling inhibits M1 polarization of macrophages, alleviates local pancreatic inflammation, and thus plays a role in preserving the functional integrity of pancreatic β cells [99,100]. Bile acids are also capable of activating the farnesoid X receptor (FXR) signaling pathway, which collaborates with other signaling cascades to regulate inflammatory responses and maintain energy metabolic balance [100]. LCA can also act as an endogenous ligand for VDR to directly activate VDR-mediated signaling. This finding implies the existence of a bidirectional regulatory feedback loop between bile acids and vitamin D [101,102]. In states of vitamin D deficiency, bile acid synthesis, transport, and microbiota-mediated transformation may become dysregulated, resulting in an abnormal proportion of secondary bile acids. This imbalance may weaken FXR- and TGR5-mediated signaling and indirectly impair VDR activation, ultimately leading to reduced GLP-1 secretion, decreased insulin sensitivity, and disruption of glucose metabolic homeostasis [50,103,104].

Trp metabolism mainly involves the kynurenine, serotonin, and indole pathways, with the indole pathway being the one most closely linked to the gut microbial ecosystem [105]. After binding to the nuclear VDR, vitamin D can directly interact with vitamin D response elements (VDREs) in the indoleamine 2,3-dioxygenase 1 (IDO1) gene promoter region to inhibit the transcription and protein expression of IDO1 [106]. Meanwhile, it competitively blocks the IDO1 activation pathway induced by inflammatory factors (e.g., IFN-γ) [107], because kynurenine metabolites may promote inflammation, oxidative stress, and β-cell damage [105]. This prevents excessive Trp shunting into the kynurenine pathway and reduces the accumulation of immunosuppressive or neurotoxic metabolites, such as 3-hydroxykynurenine and quinolinic acid [108,109]. At the same time, VDR activation upregulates the expression of intestinal epithelial mucins (e.g., MUC2) [69]. This regulatory effect helps establish a stable colonization niche for symbiotic tryptophanase-producing bacteria, such as *Lactobacillus* and *Bifidobacterium* species. It also facilitates the conversion of tryptophan into indole metabolites, including indole, indole-3-propionic acid, and indole-3-acetic acid [110,111]. These indole metabolites strengthen intestinal barrier function, decrease LPS translocation, and suppress systemic inflammatory responses by activating the aryl hydrocarbon receptor (AhR) or pregnane X receptor [84,111]. When indole derivatives bind to AhR, they induce nuclear translocation and heterodimer formation of the receptor [105]. This process upregulates the expression of downstream genes involved in anti-inflammatory responses and intestinal barrier repair, thereby alleviating intestinal inflammation and reducing systemic insulin resistance induced by endotoxemia [107,112]. In this way, vitamin D modulates T2DMby re-establishing the balance of Trp metabolic pathways.

Beyond these metabolites, vitamin D may also impact the development and progression of T2DM through other microbial metabolites. Trimethylamine N-oxide (TMAO) forms when the liver oxidizes trimethylamine (TMA). TMA is a byproduct of dietary choline and carnitine metabolism, produced by gut microbiota. Higher TMAO levels show a close link to inflammation, oxidative stress and insulin resistance [113]. Vitamin D can modify gut microbiota composition. This change reduces the abundance of bacteria that produce TMA, and in turn lowers TMAO levels in the body [114]. Similarly, elevated BCAA levels in plasma correlate positively with insulin resistance. BCAAs include leucine, isoleucine and valine. The gut microbiota have an important role in synthesizing and degrading these amino acids [115,116,117]. Vitamin D may ease the abnormal buildup of BCAAs by improving microbial homeostasis and energy metabolism. This action may also help improve T2DM symptoms [118].

## 6. Conclusions

To sum up, available evidence shows that vitamin D can regulate the integrity of the intestinal barrier, intestinal inflammation, immune responses and the profile of gut-derived metabolites through multiple pathways. In this way, it affects the host’s metabolic homeostasis of glucose and lipids, which may reduce the risk of T2DM. Among these factors, gut metabolites such as SCFAs, BAs and Trp play a key mediating role between vitamin D and metabolic phenotypes. Vitamin D deficiency may cause intestinal dysbiosis, impaired intestinal barrier function and low-grade systemic inflammation. These changes further reduce insulin sensitivity and promote metabolic disorders. In contrast, sufficient vitamin D levels help maintain the homeostasis of intestinal microecology and participate in the regulation of glucose metabolism through the above key metabolites.

However, current studies still have several limitations. These include the heterogeneity of study subjects and study designs, inconsistent methods for assessing vitamin D status and intervention protocols, as well as high diversity of outcome indicators related to intestinal microbiota. In addition, T2DM is a metabolic disease driven by multiple factors: its complex etiological characteristics, together with the limitations of extrapolating results from rodent models to human studies, have to some extent restricted the interpretability of existing evidence.

Future research needs to further identify the optimal dosage and intervention timing of vitamin D supplementation. Combined with the characteristics of individualized intestinal microbiota, researchers should explore precise metabolic regulation strategies based on the vitamin D–gut microbiota axis. Although accumulating evidence supports vitamin D as a potential adjunctive intervention for cardiometabolic diseases, its clinical translational value needs to be verified by well-designed large-scale randomized controlled trials.

## Data Availability

No new data were created or analyzed in this study. Data sharing is not applicable to this article.
